# Trends in Mortality Attributed to Heart Failure Among Older Adults in Brazil, 2000-2024

**DOI:** 10.7759/cureus.114265

**Published:** 2026-08-10

**Authors:** Fernanda L Froes, Jessica N Tsurumaki, Leonora J Ferreira, Marcela K Baronian, Fernanda S Schütz, Bianca A Cotrim, Lara R Lima Ribeiro, Vinicius F Linares, Flávio Henrique N Reges, Humberto G Moreira

**Affiliations:** 1 Faculty of Medicine, Universidade Anhembi Morumbi, Sao Paulo, BRA; 2 Medicine, Universidade Nove de Julho, São Paulo, BRA; 3 Faculty of Medicine, Pontifícia Universidade Católica de Goiás, Goiania, BRA; 4 Faculty of Medicine, Universidade de Mogi das Cruzes, Sao Paulo, BRA; 5 Medical School, Faculdade São Leopoldo Mandic, Campinas, BRA; 6 Faculty of Medicine, Universidade Cidade de São Paulo, Sao Paulo, BRA; 7 Faculty of Medicine, Universidade de Rio Verde, Rio Verde, BRA; 8 Faculty of Medicine, Anhanguera-Uniderp University, Campo Grande, BRA; 9 Faculty of Medicine, Federal University of Goiás, Goiânia, BRA; 10 Hospital Medicine, Einstein Hospital Israelita, Sao Paulo, BRA

**Keywords:** elderly, epidemiology of heart failure, epidemiology of hf, heart failure, mortality, mortality trends in brazil

## Abstract

Background: Heart failure is a leading cause of death in older adults, but long-term population-based evidence from middle-income countries remains limited. We examined national and regional trends in mortality assigned to heart failure among Brazilian adults aged 60 years or older from 2000 to 2024.

Methods: We conducted a nationwide, population-based time-series study using the Brazilian Mortality Information System. Deaths were included if the International Classification of Diseases, 10th Revision, code I50 was recorded as the underlying cause. Population denominators came from official census counts and intercensal estimates. We calculated crude, age-specific, and age-standardized mortality rates per 100,000 population using direct standardization to the age distribution of Brazilians aged 60 years or older in the 2022 census. Trends over 2000-2024 were estimated by weighted log-linear regression and expressed as annual percentage changes with 95% CIs. Regional heterogeneity was assessed with a calendar year-by-region interaction.

Results: Between 2000 and 2024, 601,490 deaths were assigned to heart failure as the underlying cause among Brazilian adults aged 60 years or older. The age-standardized mortality rate declined from 157.4 per 100,000 in 2000 to 83.7 per 100,000 in 2024, a 47% reduction (annual percentage change −2.68%, 95% CI −2.96 to −2.41). Rates declined in both sexes, all age groups, and all macroregions. Declines were fastest in the Central-West and South and slowest in the North and Northeast (p < 0.001); the North had the highest regional rate in 2024. Despite falling rates, the annual number of deaths increased from 23,213 in 2000 to 27,909 in 2024. The post-2019 plateau persisted when the pandemic years were excluded.

Conclusions: Mortality assigned to heart failure among older Brazilian adults declined markedly, but progress was uneven, and the absolute number of deaths rose as the population aged. Slower declines in the North and Northeast and the persistent burden among the oldest adults identify priorities for surveillance and health-system planning.

## Introduction

Heart failure is a major cause of death and health-care use worldwide, and its burden is increasingly concentrated in older adults [1]. Across low-income and middle-income countries, outcomes vary with underlying cardiovascular disease profiles, access to care, and health-system capacity [2]. Evidence from Latin America remains limited but indicates substantial mortality and recurrent hospital use [3].

Brazil is aging rapidly. The population aged 60 years or older more than doubled between 2000 and 2024, from approximately 15.2 million to 34.2 million people, and exceeded 32 million in the 2022 census [4]. Brazilian studies have documented poor outcomes after acute heart failure, gaps in evidence-based care, declining mortality since the late 1990s, and marked geographic variation [5-7]. However, previous national analyses did not extend through 2024 or focus exclusively on older adults using the 2022 census age distribution for standardization.

We examined mortality assigned to heart failure among Brazilian adults aged 60 years or older from 2000 to 2024. The prespecified primary objective was to estimate the national trend in age-standardized mortality. Secondary objectives were to assess trends by sex, age group, and macroregion. We hypothesized that mortality would decline overall but remain heterogeneous across regions.

## Materials and methods

Study design and data sources

We conducted a nationwide, population-based ecological time-series study of deaths assigned to heart failure among adults aged 60 years or older in Brazil from January 1, 2000, to December 31, 2024.

Mortality data were obtained from the Brazilian Mortality Information System through DATASUS/TabNet on July 1, 2026. The system compiles death certificate data, including the underlying cause, age, sex, year of death, and region of residence [8].

Population denominators were obtained from official census counts and intercensal estimates produced by the Brazilian Institute of Geography and Statistics, stratified by year, sex, age group, and macroregion. For the supplementary assessment of ascertainment, we also extracted deaths among adults aged 60 years or older assigned to ill-defined symptoms and findings, ill-defined circulatory diseases, or ill-defined respiratory diseases.

The study was reported in accordance with the REporting of studies Conducted using Observational Routinely-collected health Data (RECORD) Statement and the Guidelines for Accurate and Transparent Health Estimates Reporting [9,10].

Study population

We included all deaths recorded among Brazilian residents aged 60 years or older for which heart failure was selected as the underlying cause. This threshold aligns with the definition of older adults used in Brazilian demographic and public health policy. Heart failure was defined as International Classification of Diseases, 10th Revision, category I50, including congestive heart failure (I50.0), left ventricular failure (I50.1), and heart failure, unspecified (I50.9) [11]. Deaths in which heart failure appeared only as an associated or contributing cause were not included.

Age was defined at death and macroregion by place of residence. Deaths with unknown age were excluded because eligibility could not be established. Deaths with unknown sex were retained in national and regional analyses but excluded from sex-specific analyses. The study included all eligible deaths recorded in the national system; no sample size calculation was required.

Outcomes

The primary outcome was the annual age-standardized mortality rate assigned to heart failure as the underlying cause per 100,000 residents aged 60 years or older.

Secondary outcomes were the annual number of deaths, crude mortality rates, age-specific mortality rates, and regional age-standardized mortality rates. Age-specific rates were calculated for ages 60-64, 65-69, 70-74, 75-79, and 80 years or older.

Mortality rates were calculated by dividing the number of eligible deaths in each calendar year and geographic stratum by the corresponding resident population and multiplying the result by 100,000.

Age standardization

Mortality rates were age-standardized by the direct method using the age distribution of the Brazilian population aged 60 years or older in the 2022 census as the standard population [4].

For each calendar year and geographic region, age-specific mortality rates were multiplied by the corresponding proportions of the standard population. The weighted age-specific rates were then summed to obtain the age-standardized mortality rate.

The same standard population and age categories were used for all national and regional estimates, ensuring comparability over time and between regions. Crude rates were retained as secondary measures because they represent the observed population burden, whereas age-standardized rates were used for temporal and regional comparisons.

Statistical analysis

Annual deaths and crude, age-specific, and age-standardized mortality rates were summarized with 95% CIs. Poisson CIs were used for crude and age-specific rates, and the Fay-Feuer gamma method was used for directly standardized rates.

Temporal trends were estimated using inverse-variance weighted segmented log-linear regression of annual age-standardized rates on calendar year. Candidate models with different numbers and locations of breakpoints were fitted, and the final specification was selected using the Bayesian information criterion. Breakpoints were therefore estimated from the observed series rather than imposed a priori.

Within each segment, the annual percentage change was calculated as 100 × (exp(β_k) − 1), where β_k represents the corresponding log-linear slope. Confidence intervals were derived from the relevant linear combinations of the dispersion-scaled covariance matrix. Trends were classified as increasing or decreasing when the 95% CI excluded zero and as stable when it included zero.

National, sex-specific, and regional trends were estimated separately. Regional heterogeneity was assessed in a pooled model containing calendar year, macroregion, and their interaction. Complementary negative binomial models used annual deaths as the outcome and the logarithm of the corresponding population as an offset. Overdispersion was assessed before fitting these models, and negative binomial specifications were adopted accordingly.

Residual autocorrelation was assessed using autocorrelation plots and the Durbin-Watson and Ljung-Box tests. Heteroscedasticity- and autocorrelation-consistent standard errors were prespecified for use if significant residual autocorrelation was detected.

Sensitivity analyses used crude rates, Poisson count models, and the exclusion of deaths recorded during 2020-2021. No missing values were imputed. All tests were two-sided, with a significance level of 0.05. Analyses were performed using R version 4.6.1 (R Foundation for Statistical Computing, Vienna, Austria).

Ethical considerations

The study used aggregated, publicly available data without individual identifiers and did not require research ethics review or informed consent under Brazilian regulations. Review by a research ethics committee and individual informed consent were therefore not required. Patients and members of the public were not involved in the design, conduct, analysis, or reporting of the study.

## Results

Deaths and the study population

Between January 1, 2000, and December 31, 2024, 601,490 eligible deaths were recorded. An additional 528 deaths with ICD-10 category I50 had unknown age and were excluded. Among the included deaths, 93 had unknown sex and were excluded only from sex-specific analyses. Because the analysis included the complete population of eligible deaths, no missing values were imputed (Table 1).

**Table 1:** Annual number of deaths and crude and age-standardized mortality rates assigned to heart failure as the underlying cause among adults aged 60 years or older, Brazil, 2000–2024. Both sexes. Rates are expressed per 100,000 residents aged ≥60 years. Age-standardized rates were calculated by the direct method using the 2022 Brazilian census population aged 60 years or older as the standard population. ASR (age-standardized rate) and CI (confidence interval) are reported. Confidence intervals were calculated using the exact Poisson method for crude and age-specific rates and the Fay-Feuer gamma method for age-standardized rates.

Year	No. of deaths	Crude rate	Crude rate 95% CI	Age-standardized rate	ASR 95% CI
2000	23,213	152.4	150.5–154.4	157.4	155.4–159.8
2001	22,541	144.0	142.2–145.9	148.4	146.4–150.7
2002	22,706	141.2	139.4–143.1	144.9	143.1–147.2
2003	22,660	137.2	135.4–139.0	140.1	138.3–142.3
2004	22,954	135.1	133.4–136.9	137.4	135.6–139.6
2005	22,214	126.9	125.2–128.6	128.5	126.8–130.6
2006	23,456	129.8	128.2–131.5	131.0	129.3–133.1
2007	23,615	126.5	124.9–128.1	127.4	125.8–129.5
2008	23,262	120.4	118.9–122.0	121.2	119.6–123.2
2009	23,204	116.0	114.5–117.5	116.8	115.3–118.7
2010	23,464	113.3	111.8–114.7	114.1	112.7–116.0
2011	23,820	110.9	109.5–112.3	111.9	110.5–113.8
2012	22,796	102.3	101.0–103.7	103.5	102.1–105.3
2013	23,458	101.5	100.2–102.8	102.8	101.5–104.6
2014	23,213	96.7	95.5–98.0	98.2	96.9–99.9
2015	23,833	95.6	94.4–96.9	97.2	96.0–98.9
2016	24,878	96.2	95.0–97.4	97.9	96.7–99.6
2017	24,047	89.5	88.4–90.7	91.2	90.0–92.8
2018	23,044	82.6	81.6–83.7	84.2	83.1–85.7
2019	23,659	81.7	80.7–82.8	83.3	82.2–84.8
2020	24,099	80.4	79.4–81.4	82.0	81.0–83.5
2021	27,314	88.4	87.3–89.4	90.5	89.4–92.0
2022	29,039	91.1	90.1–92.2	93.7	92.6–95.2
2023	27,092	82.1	81.2–83.1	84.5	83.5–86.0
2024	27,909	81.7	80.7–82.6	83.7	82.7–85.2

National trends in age-standardized mortality (primary outcome)

**Figure 1:**
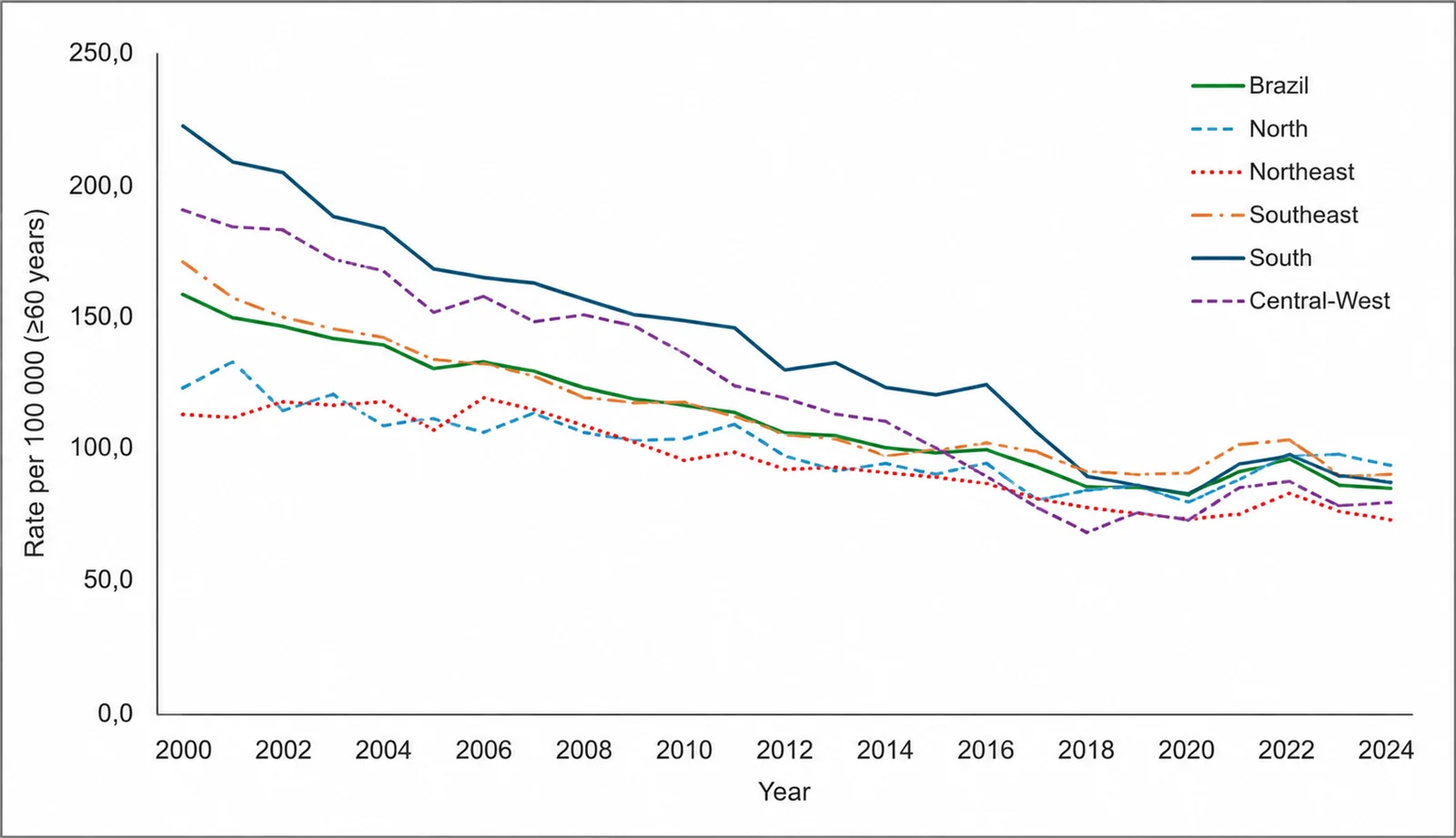
Age-standardized mortality rates assigned to heart failure among adults aged 60 years or older, by macroregion, Brazil, 2000–2024. Rates are per 100,000 residents and were directly age-standardized to the 2022 Brazilian census population.

Nationally, the age-standardized mortality rate assigned to heart failure as the underlying cause fell from 157.4 (95% CI 155.4-159.8) deaths per 100,000 residents aged 60 years or older in 2000 to 83.7 (82.7-85.2) per 100,000 in 2024, a relative reduction of 47% over the 25 calendar years studied (Table 1, Figure 1). The crude rate followed the same trajectory, from 152.4 to 81.7 per 100,000. The pace of that decline was not constant. Segmented regression identified a single breakpoint at 2019.

The segmented log-linear model selected by the Bayesian information criterion included a single breakpoint in 2019. Mortality declined by 3.10% per year between 2000 and 2019 (95% CI −3.32 to −2.88) and showed no significant change between 2019 and 2024 (APC 0.49%, 95% CI −0.60 to 1.59).

Trends by sex

Age-standardized mortality declined in both sexes over 2000-2024. The full-period APC was −2.58% in men (95% CI −2.85 to −2.31) and −2.76% in women (95% CI −3.05 to −2.47; Table 2). The male-to-female rate ratio increased from 1.03 in 2000 to 1.09 in 2024, while the absolute difference increased from 4.2 to 7.4 deaths per 100,000.

**Table 2:** Age-standardized mortality rates assigned to heart failure in 2000 and 2024 and annual percentage change over 2000–2024, by macroregion and sex, adults aged 60 years or older, Brazil. Rates are expressed per 100,000 residents aged 60 years or older and were age-standardized by the direct method using the 2022 Brazilian census population as the standard population. APC (annual percentage change) was estimated over 2000-2024 using weighted log-linear regression as 100 × (exp(β) − 1), where β is the log-linear slope for calendar year. ASR denotes the age-standardized rate. Regional heterogeneity was assessed using a calendar year-by-macroregion interaction (p < 0.0001).

Macroregion	Sex	ASR 2000	ASR 2024	APC (%/year)	95% CI
Brazil	Both	157.4	83.7	-2.68	-2.96 to -2.41
Brazil	Male	159.6	87.8	-2.58	-2.85 to -2.31
Brazil	Female	155.4	80.4	-2.76	-3.05 to -2.47
North	Both	120.7	92.3	-1.48	-1.89 to -1.07
North	Male	130.4	95.7	-1.65	-2.05 to -1.25
North	Female	109.4	88.2	-1.24	-1.72 to -0.75
Northeast	Both	111.5	72.9	-2.13	-2.42 to -1.84
Northeast	Male	120.0	81.2	-2.01	-2.29 to -1.72
Northeast	Female	103.1	66.1	-2.21	-2.52 to -1.89
Southeast	Both	169.8	88.7	-2.48	-2.84 to -2.11
Southeast	Male	171.8	91.4	-2.39	-2.74 to -2.04
Southeast	Female	168.5	86.8	-2.54	-2.93 to -2.14
South	Both	221.5	86.4	-4.03	-4.39 to -3.66
South	Male	215.8	88.3	-3.91	-4.32 to -3.50
South	Female	225.7	84.9	-4.12	-4.47 to -3.76
Central-West	Both	188.9	78.6	-4.29	-4.80 to -3.77
Central-West	Male	186.4	82.9	-4.27	-4.79 to -3.74
Central-West	Female	189.3	74.3	-4.27	-4.83 to -3.70

Age-specific trends

**Figure 2:**
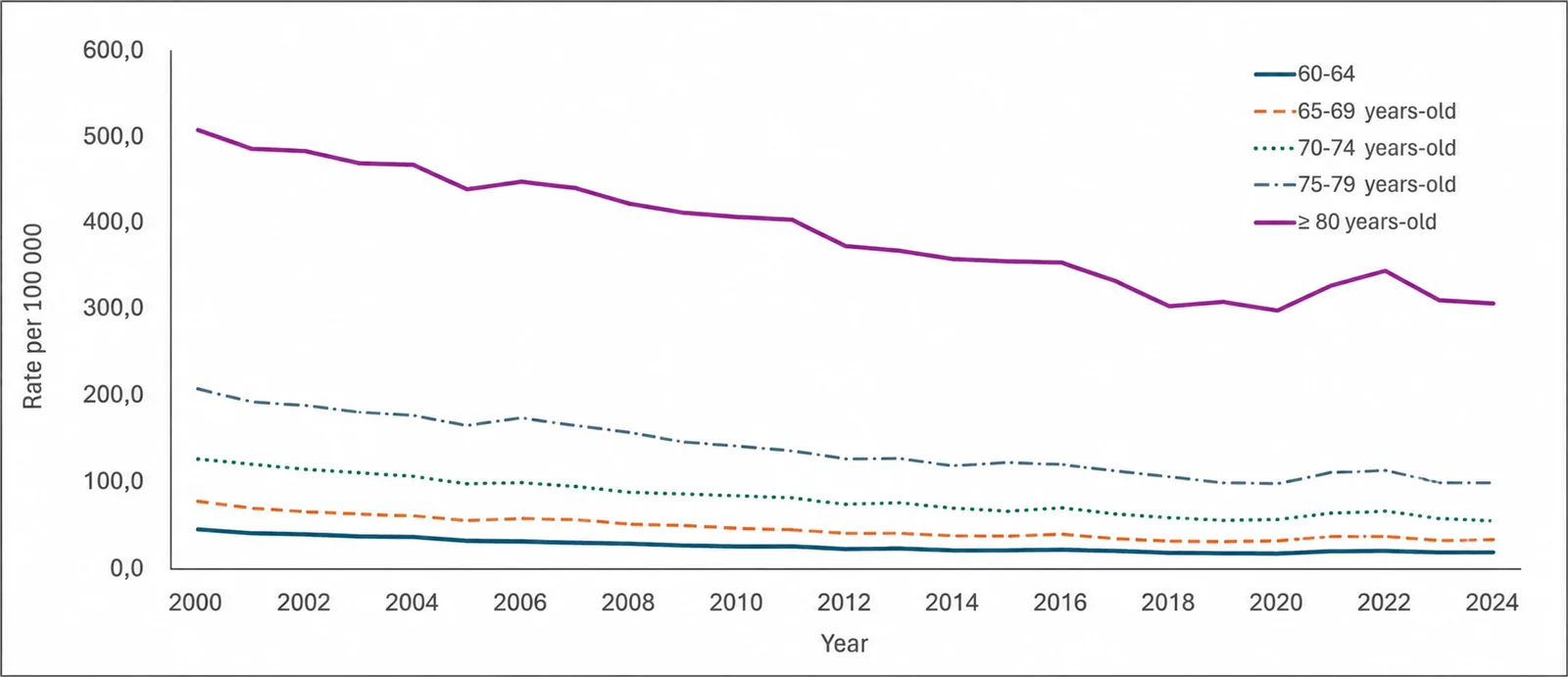
Age-specific mortality rates assigned to heart failure among adults aged 60 years or older, Brazil, 2000–2024. Rates are shown per 100,000 residents for both sexes combined.

Mortality declined across every age group between 2000 and 2024 (Figure 2). Age-specific rates fell from 48.5 to 21.1 per 100,000 at 60-64 years, from 82.0 to 35.7 at 65-69 years, from 133.5 to 56.6 at 70-74 years, from 229.4 to 101.0 at 75-79 years, and from 562.4 to 282.4 at 80 years or older. The relative decline was smallest in the oldest adults (49.8% at ≥80 years) and largest in the youngest (56.5% at 60-64 years). Consequently, the age gradient widened rather than narrowed: mortality at 80 years or older was 11.6 times that at 60-64 years in 2000 and 13.4 times in 2024.

Regional trends (secondary outcome)

Age-standardized mortality declined in all macroregions (Table 2, Figure 1). Regional rates narrowed from a 2.0-fold range in 2000 to 1.3-fold in 2024. Annual declines were largest in the Central-West (−4.29%, 95% CI −4.80 to −3.77) and South (−4.03%, 95% CI −4.39 to −3.66), and smallest in the North (−1.48%, 95% CI −1.89 to −1.07) and Northeast (−2.13%, 95% CI −2.42 to −1.84). The North had the highest regional rate in 2024. Regional trends differed significantly from one another. In a pooled model containing calendar year, region, and a calendar year × region interaction, the interaction term was highly significant (p < 0.0001), confirming that the pace of decline was not uniform across regions (Table 2).

Sensitivity analyses

Sensitivity analyses produced similar estimates (Appendices). The full-period APC was −2.70% using crude rates (95% CI −2.96 to −2.44) and −2.69% after excluding deaths recorded in 2020-2021 (95% CI −2.97 to −2.40). Restricting the post-2019 segment to 2019 and 2022-2024, excluding the pandemic years 2020-2021, yielded an APC of 0.17% (95% CI −7.41 to 8.38; Appendices), consistent with the primary post-2019 estimate (APC 0.49%, 95% CI −0.60 to 1.59), though imprecise given the small number of non-consecutive years available. Sex-specific and regional conclusions were unchanged. No significant residual autocorrelation was detected in the national, sex-specific, or regional models using autocorrelation plots and the Durbin-Watson and Ljung-Box tests (all p > 0.10). HAC standard errors were therefore not required.

## Discussion

Mortality assigned to heart failure among older adults in Brazil declined over the study period, but the gains were uneven. The regions with the highest rates at the outset improved most rapidly, whereas progress was slower in the North and Northeast. This changed the geographic distribution of mortality and left the North with the least favorable position by the end of the series. At the same time, population aging sustained the absolute burden despite lower mortality rates.

Earlier Brazilian studies described a national decline alongside substantial spatial variation. Arruda and colleagues found a less favorable trajectory in the North [6], and Cestari and colleagues identified clusters of higher mortality in the North and Northeast [7]. The present analysis extends those observations to a contemporary cohort of older adults and uses the 2022 census population for standardization. The regional convergence seen over time should therefore not be read as uniform progress. It reflects rapid improvement in regions with a high initial burden and slower gains elsewhere.

The national decline is compatible with advances in cardiovascular prevention and treatment, although the ecological design does not allow the contribution of individual mechanisms to be determined. Socioeconomic development has been associated with heart failure mortality in Brazil, and broader coverage by the Family Health Strategy has been linked to fewer heart failure admissions in São Paulo state [12,13]. Regional differences in cardiologist density, access to specialized heart failure services, diagnostic testing, advanced cardiovascular therapies, primary care, hospital infrastructure, and socioeconomic conditions may also have contributed. These factors were not directly measured and should therefore be considered plausible structural explanations rather than demonstrated causes.

The Brazilian pattern is consistent with broader evidence from middle-income settings. International Congestive Heart Failure study (INTER-CHF) showed marked regional differences in prognosis after adjustment for clinical characteristics [14], and International REgistry to assess medical Practice with lOngitudinal obseRvation for Treatment of Heart Failure (REPORT-HF) linked post-discharge outcomes to national income and income inequality [15]. Although these cohorts assessed hospital and post-discharge outcomes rather than population mortality, they illustrate how strongly heart failure prognosis depends on health-system organization and access to care.

Population mortality and hospital case fatality describe different stages of the disease. Brazilian administrative data show fewer admissions for heart failure over time but higher mortality among those admitted [16], while BREATHE (Brazilian Registry of Heart Failure) and tertiary-care cohorts continue to document substantial early mortality, readmission, and clinical complexity [17,18]. One possible explanation is that patients who reach the hospital now represent a more selected group with advanced disease, frailty, and multimorbidity. The present data cannot test that hypothesis.

The oldest adults remained disproportionately affected and experienced smaller relative gains than younger groups. This pattern is consistent with the accumulation of structural heart disease, renal dysfunction, frailty, and competing illness with advancing age [17,18]. It also has practical consequences: as the population aged 80 years or older expands, demand for integrated cardiovascular and geriatric care will continue to grow even if age-specific mortality keeps falling.

The temporary rise in recorded mortality during the COVID-19 period did not materially alter the long-term estimate. Disrupted outpatient care, delayed presentation, SARS-CoV-2 infection, and changes in death certification could all have contributed to this short-term fluctuation. The study was not designed to distinguish among these mechanisms or estimate a causal pandemic effect. The post-2019 plateau was not attributable to the pandemic years alone, suggesting prolonged disruption of chronic heart failure care, lingering cardiovascular effects of SARS-CoV-2 infection, or diminishing gains from current management strategies. These explanations remain speculative and warrant further investigation.

One specific caveat applies to the regional comparison. Over the same period, the quality of cause-of-death certification improved throughout Brazil: deaths assigned to ill-defined causes fell from 145,051 in 2000 to 77,987 in 2024, a reduction of 46% (Appendices). Improved ascertainment may have shifted deaths from ill-defined categories to specific causes, including heart failure, thereby attenuating the apparent decline in the North and Northeast. Because the Northeast showed both the greatest improvement in certification and one of the slowest declines in heart failure mortality, part of the observed regional convergence may reflect differential ascertainment rather than true convergence in underlying risk. As ill-defined causes were not redistributed, the magnitude of this effect cannot be quantified.

This study has several strengths. It used nationwide population-based mortality data covering 25 consecutive years and included all eligible deaths recorded in the Brazilian Mortality Information System. Rates were standardized to the age distribution of the population aged 60 years or older in the 2022 Brazilian census, providing a contemporary and internally consistent basis for comparisons over time and across regions. The analysis also included prespecified assessments by age, sex, and macroregion, together with sensitivity analyses that supported the robustness of the main findings.

Several limitations should be acknowledged. First, the outcome was restricted to deaths for which heart failure was selected as the underlying cause. Heart failure often lies within the causal pathway to death rather than being chosen as the underlying cause. A Brazilian multiple-cause study found that heart failure was mentioned on death certificates substantially more often than it was selected as the underlying cause [19]. The present findings, therefore, describe mortality formally assigned to heart failure rather than the full mortality burden among people living with the syndrome. Changes in diagnostic practice and death certification may also have influenced the observed trends. Second, aggregated data did not permit assessment of heart failure phenotype, etiology, severity, comorbidities, race or ethnicity, socioeconomic circumstances, treatment, or access to care. Finally, the ecological design precludes individual-level and causal inference.

## Conclusions

Mortality assigned to heart failure among older adults in Brazil declined substantially over the past quarter-century, but progress remained uneven across regions and age groups. The increasing number of deaths despite declining age-standardized mortality reflects rapid population aging rather than worsening age-adjusted risk. Slower declines in the North and Northeast and the persistent concentration of mortality among the oldest adults identify priorities for further epidemiologic investigation and health system planning.

## Appendices

**Table 3:** Sensitivity analyses with annual percentage change (95% CI) under alternative model specifications, by region and sex. APC (annual percentage change) was derived from log-linear regression as 100 × (exp(β) − 1), where β is the slope of ln(rate) on calendar year. The Primary (ASR) column presents results using age-standardized rates for the full study period (2000-2024). Sensitivity 1 (crude) presents results using crude mortality rates for the full period. Sensitivity 2 (excl. 2020-21) presents results using age-standardized rates after excluding the pandemic years 2020-2021 (2000-2019 and 2022-2024). Sensitivity 3 (post-2019, excl. 2020-21) presents results from the post-2019 segment only (2019 and 2022-2024, excluding 2020-2021) of a two-segment (joinpoint) model anchored at 2019 to assess whether the post-2019 trend persisted after the pandemic years were excluded. Trends were classified as decreasing (or increasing) when the 95% CI excluded zero and as stable when the 95% CI included zero. All models excluded individuals younger than 60 years.

Region	Sex	Primary (ASR)	Sensitivity 1 (crude)	Sensitivity 2 (excl. 2020–21)	Sensitivity 3 (post-2019, excl. 2020–21)
Brazil	Both	-2.68 (-2.96 to -2.41)	-2.70 (-2.96 to -2.44)	-2.69 (-2.97 to -2.40)	0.17 (-7.41 to 8.38)
Brazil	Male	-2.58 (-2.85 to -2.31)	-2.63 (-2.91 to -2.36)	-2.59 (-2.87 to -2.30)	0.62 (-5.43 to 7.06)
Brazil	Female	-2.76 (-3.05 to -2.47)	-2.75 (-3.01 to -2.48)	-2.77 (-3.07 to -2.46)	-0.18 (-9.09 to 9.60)
North	Both	-1.48 (-1.89 to -1.07)	-1.80 (-2.22 to -1.38)	-1.41 (-1.84 to -0.97)	2.34 (-3.45 to 8.49)
North	Male	-1.65 (-2.05 to -1.25)	-1.88 (-2.29 to -1.47)	-1.62 (-2.06 to -1.18)	1.58 (-5.09 to 8.72)
North	Female	-1.24 (-1.72 to -0.75)	-1.66 (-2.15 to -1.16)	-1.12 (-1.62 to -0.63)	3.33 (-1.52 to 8.41)
Northeast	Both	-2.13 (-2.42 to -1.84)	-2.24 (-2.53 to -1.94)	-2.06 (-2.37 to -1.75)	-0.41 (-7.61 to 7.36)
Northeast	Male	-2.01 (-2.29 to -1.72)	-2.22 (-2.52 to -1.92)	-1.95 (-2.26 to -1.63)	0.34 (-6.10 to 7.23)
Northeast	Female	-2.21 (-2.52 to -1.89)	-2.22 (-2.54 to -1.90)	-2.12 (-2.44 to -1.79)	-1.07 (-9.03 to 7.58)
Southeast	Both	-2.48 (-2.84 to -2.11)	-2.37 (-2.68 to -2.05)	-2.56 (-2.93 to -2.18)	0.07 (-8.54 to 9.49)
Southeast	Male	-2.39 (-2.74 to -2.04)	-2.30 (-2.63 to -1.97)	-2.47 (-2.82 to -2.11)	0.09 (-6.95 to 7.66)
Southeast	Female	-2.54 (-2.93 to -2.14)	-2.42 (-2.74 to -2.11)	-2.62 (-3.04 to -2.21)	0.06 (-9.83 to 11.04)
South	Both	-4.03 (-4.39 to -3.66)	-3.95 (-4.31 to -3.58)	-3.95 (-4.31 to -3.59)	0.31 (-6.43 to 7.54)
South	Male	-3.91 (-4.32 to -3.50)	-3.84 (-4.25 to -3.42)	-3.83 (-4.22 to -3.44)	1.55 (-1.73 to 4.94)
South	Female	-4.12 (-4.47 to -3.76)	-4.03 (-4.39 to -3.67)	-4.05 (-4.41 to -3.68)	-0.61 (-10.17 to 9.96)
Central-West	Both	-4.29 (-4.80 to -3.77)	-4.27 (-4.78 to -3.75)	-4.28 (-4.83 to -3.72)	0.68 (-6.61 to 8.53)
Central-West	Male	-4.27 (-4.79 to -3.74)	-4.18 (-4.69 to -3.65)	-4.24 (-4.81 to -3.67)	2.27 (-3.68 to 8.59)
Central-West	Female	-4.27 (-4.83 to -3.70)	-4.33 (-4.89 to -3.76)	-4.27 (-4.87 to -3.67)	-0.83 (-9.66 to 8.86)

**Table 4:** Annual number of deaths assigned to ill-defined causes, by macroregion, Brazil, 2000–2024. Ill-defined causes comprise ICD-10 Chapter XVIII (symptoms, signs, and abnormal clinical and laboratory findings, excluding sudden infant death), together with ill-defined circulatory and ill-defined respiratory causes. Data are presented for all ages and both sexes, by year of death and region of residence. The final row shows the relative change between 2000 and 2024.

Year	North	Northeast	Southeast	South	Central-West	Brazil
2000	11,840	67,526	50,089	10,558	5,038	145,051
2001	12,049	68,537	49,751	10,483	4,863	145,683
2002	11,363	69,829	47,966	10,793	4,272	144,223
2003	11,635	68,299	47,560	11,470	3,702	142,666
2004	11,689	63,641	45,320	11,179	3,952	135,781
2005	10,232	46,647	41,652	10,172	3,599	112,302
2006	8,709	25,974	42,323	10,072	3,202	90,280
2007	7,570	22,801	41,685	9,919	2,881	84,856
2008	8,060	23,244	40,025	9,183	2,887	83,399
2009	8,241	23,170	39,321	9,471	2,763	82,966
2010	8,166	23,599	39,773	9,241	3,016	83,795
2011	7,935	24,984	38,013	9,007	3,412	83,351
2012	8,009	24,440	35,762	9,098	2,597	79,906
2013	6,775	24,840	34,404	8,464	2,198	76,681
2014	6,471	25,301	33,945	7,936	2,415	76,068
2015	6,650	26,374	34,114	7,272	2,613	77,023
2016	6,677	27,226	36,372	8,642	2,744	81,661
2017	6,269	25,010	36,108	7,698	2,687	77,772
2018	6,679	22,426	37,040	7,915	2,407	76,467
2019	7,578	24,269	38,638	8,000	2,930	81,415
2020	9,325	30,096	47,431	8,602	3,453	98,907
2021	8,221	29,537	52,563	9,696	3,402	103,419
2022	7,299	27,710	44,698	9,447	3,237	92,391
2023	6,535	22,702	40,590	7,903	2,988	80,718
2024	6,561	20,754	39,525	7,980	3,167	77,987
Change 2000–2024	-45%	-69%	-21%	-24%	-37%	-46%
